# The Need for Greater Uniformity in the Division of the Abdominal Regions

**Published:** 1893-09

**Authors:** 


### THE NEED FOR GREATER UNIFORMITY IN THE DIVISION OF THE ABDOMINAL REGIONS.

It is as true of sciences as of habits, that if we begin with a foundation in which everything about it is not exact and mathematically accurate, so to say, the superstructure will reveal similar defects. This is strikingly so in the science of anatomy, in which the size, position, etc., of the various organs and portions of the organism vary in each individual case, and, therefore, do not admit of being described with mathematical precision. Our estimates are only approximate. The result has been that in their descriptions of regions or organs authors show a laxity, begotten, it is true, by the nature of the subject, but in which they have gone much farther in the approximative than seems justifiable. This is the more easy in a field of science such as anatomy, where originality is not easily shown, and the desire to make one's name live by putting this line in one place and that in another (in the majority of instances but a few millimeters from the place of someone else), is readily yielded to. Unless by so doing one simplifies matters and seeks to bring about such a condition as shall lead to uniformity, it is better to desist. These remarks have been suggested by the lack of uniformity shown by various authors in marking out the regions of the abdomen. There are over one dozen methods of placing lines upon the surface of the abdomen, thus dividing it into regions, so that an organ situated in one region, according to one author, is found either not at all, or only in part in the same region according to a second. Confusion is the natural result, and a science which should supply the necessary to the daily needs of the practitioner does not do so at all.

In a communication to the Anatomical Society of Great Britain, in May last, Professor Anderson made a strong plea for a greater uniformity and simplification in setting the limits of the abdominal regions, and suggested a plan which, to our mind, is a great improvement upon anything heretofore advanced. His plan is briefly as follows: Draw a line from the symphysis pubis to the ensiform appendix, and draw another at right angles to this at the level of the umbilicus. This marks off the abdomen into four regions whose boundaries are fairly constant. They can be designated as upper and lower, right and left quadrants. Such regions can be demonstrated upon the dorsum with equal ease, as there the vertebral column corresponds to the vertical line anteriorly, and a line drawn between the third and fourth lumbar vertebræ, if prolonged, would join the anterior horizontal line. When the term "line" is used, it must be remembered that we are really speaking of planes of which these "lines" are but the outer borders. These regions, therefore, are so many compartments (pigeon-holes, if you will), in which the various abdominal organs are stored. Professor Anderson would reduce these from nine to four, in which the organs found would be as constant as it is possible to have them. We hope that the Association of American Anatomists may take this subject into consideration at their next meeting, and adopt this or some equally simple and excellent plan.

